# Experiences With Developing and Using Vital Sign Telemonitoring to Support Mobile Nursing in Rural Regions: Feasibility and Usability Study

**DOI:** 10.2196/17113

**Published:** 2020-04-23

**Authors:** Marco Schweitzer, Lukas Huber, Thilo Gorfer, Alexander Hörbst

**Affiliations:** 1 Institute for Biomedical Computer Sciences and Mechatronics UMIT - Private University for Health Sciences, Medical Informatics and Technology Hall in Tyrol Austria; 2 Medical Technologies Department MCI Management Center Innsbruck Innsbruck Austria

**Keywords:** mobile health, telemedicine, vital signs, health monitoring, mobile nursing

## Abstract

**Background:**

Modern information and communication technology has the potential to support mobile care in rural regions such as the Alpine region, which is characterized by long distances or even physically unreachable areas.

**Objective:**

This study investigated the potential of supporting mobile nursing organizations in rural regions with the use of mobile telemonitoring systems in a case study setting.

**Methods:**

As a subproject of the European Union–funded project INTESI, the VITAl parameter MOnitoring (VITAMO) project gathered stakeholders’ requirements for telemonitoring support of mobile care in rural regions and then developed and implemented a prototype system that was used for a 3-month test period with a local nursing organization in Austria. Log analysis, surveys, and interviews were used to evaluate the system according to the Technology Acceptance Model. The focus was technology assessment and user satisfaction of both patients and nurses.

**Results:**

Participants were provided Bluetooth devices to measure blood pressure, body weight, and blood glucose and to track activity. They also received a tablet with a mobile internet connection to see the results. The nurses were able to access the results remotely. Regularly executed speed tests and log analysis demonstrated the availability of high-speed mobile internet in the rural test region. Log analysis, surveys, and interviews revealed the suitability of the technology environment and showed that the system was easy to use and potentially useful. The perceived usefulness for supporting mobile care was rated meaningfully low, and the frequency of nurses using the tool declined continuously over the field test period. Further group discussions investigated this issue.

**Conclusions:**

While the technology environment with mobile internet, Bluetooth devices, and smart vital sign monitoring devices was adequate and suitable to support mobile nursing in rural regions, the potential benefit for the nursing organization could not be confirmed. Further analysis revealed that operational care processes did not follow a well-defined care strategy. Technology has the potential to leverage the available environment for developing meaningful solutions. These experiences could contribute to further investigations that need to identify and analyze existing mobile care processes at an organizational level.

## Introduction

Due to demographic changes, the amount of people receiving home care has recently increased, while the overall working population has declined. These social changes have had a significant impact on the economy and especially the health care domain. In 2016, 455,000 people in Austria received a care allowance [1]. Of these people, 147,000 (~1.7% of Austria’s total population) received mobile care [2]. These general developments in health care expenditure are also reflected in the cost of mobile care in Austria, which increased from €489.3 million in 2011 to €615.5 million in 2016 [2].

Nonetheless, as part of high-quality health care delivery, mobile care and social care are providing the necessary support for the elderly to live at home with maximum autonomy and health. The Alpine region represents a challenging environment for the provision of mobile nursing services [3]. It is characterized by rural regions with long distances or even physically unreachable regions due to weather conditions like snow in the winter. These circumstances are affecting mobile care organizations, impeding seamless home health care delivery for patients. The aim of mobile nursing or mobile care is to provide health care services for patients at their homes. These services are comprised of monitoring health conditions and status as well as conducting care activities, supporting medication and personal hygiene needs, advising the patient and family members, and documenting the conducted activities. Patients who receive home care usually have age-related chronic conditions and are visited regularly by a nurse (ie, about every 2-7 days depending on the health condition and situation).

Modern information and communication technology (ICT) could help overcome certain issues with the provision of mobile care. With the proliferation of smartphones and mobile internet, the age of the Internet of Things has started, where everyday things are connected and exchange data seamlessly and continuously [4]. Fitness trackers, smart watches, smart weight scales, and blood glucose monitors are just examples of potential hardware to enable telemonitoring applications.

Although geographical barriers for mobile care in the Alpine region are common, the overall technology infrastructure in rural regions in Austria often conforms to a high standard. According to a report by OpenSignal, Inc [5], people in Austria had access to mobile internet with 3G and 4G connections at an average download speed of 19.39 Mbps as of February 2017, ranking Austria within the top 15 countries in the world [6]. The 4G mobile connection had an availability of 75.60% with an average download speed of 26.91 Mbps as of February 2018.

The European Union–funded Alpine Space project Integrated territorial strategies for Services of General Interest (INTESI), conducted from 2014-2020, is assuring the delivery of long-term service of general interest (SGI) via integrated, territorial strategies and policies [7]. A subproject of INTESI was dedicated to investigating the potential to support mobile care for rural regions in the Alpine region, especially regarding the monitoring of vital signs. This was based on recent studies that demonstrated the importance of continuous vital sign monitoring in older age, especially of persons with chronic conditions [8]. The project was started in 2017 as a joint effort by different stakeholders, including clients, nurses, nursing scientists, health information scientists, and administration, and ended in the second quarter of 2018.

The following objectives were defined in the project:

1. Identification of technical and user-related requirements and use case scenarios for mobile and social care with a high level of client involvement
2. Conceptualization and implementation of a prototype application for vital parameter self-measurement that is integrated into nursing processes
3. Implementation of a suitable prototype application and test in the field
4. Evaluation of ICT support for mobile care related to its acceptance and impact on nursing process support

The Reutte region in Tyrol, Austria consists of 37 municipalities and was selected as the test region for this project. Tyrol is an Austrian province with a population of 750,000 people and 45 health care organizations providing home nursing and mobile care [9].

The nursing organization SGS Reutte in Tyrol was selected for the present study. The organization frequently supported 381 patients in 2018. In the same year, the mean effort for the care-related activities of a patient was 80.8 hours per year. From these, 16.9 hours per patient were spent driving to the patients‘ homes.

In the VITAl parameter MOnitoring (VITAMO) project, a system for telemonitoring vital signs was conceptualized and implemented as a prototype. The aim of the current study was focused on the field test of the prototype, final technical and user acceptance evaluation, and system’s impact as a supportive system for mobile care in the Alpine region. Thus, the paper is structured as follows: We describe the methodology by describing the overall idea of the prototype, the setting of the field test, and the methods for quantitative and qualitative evaluation, and we provide the qualitative and quantitative results on system usage, environment analysis, and user satisfaction.

## Methods

To reach the proposed goal, the prototype system for VITAMO was developed as a case study, incorporating smart devices to self-monitor the vital parameters of blood pressure, blood glucose, body weight (scale), and activity and sleep (movement tracker) as well as a tablet with mobile internet to synchronize measurements with the server. The tablet also provided a communication interface for telephone calls between the patient and nurse as well as a patient reminder function set by the nurses.

The initial step of the project involved gathering technical and user-related requirements, which included state-of-the-art transmission technology, avoiding complex patient involvement for the measurements, and adherence to existing nursing processes. Suitable use cases were derived from the gathered requirements. More details on the requirements engineering [10] and prototype development [11] have been reported previously. The prototype development followed an agile approach [12], which continuously delivered updates during the test period.

The project was approved and granted by the university ethics committee RCSEQ at the University for Health Sciences, Medical Informatics, and Technology in Austria.

### Field Test

A 3-month field test was planned and conducted with the local mobile nursing organization responsible for the selected test region Reutte. The organization determined nurses to participate in the project and use the system within their working routine. Suitable patients were recruited as study participants by the nurses, based on the criteria of physical and cognitive ability to use the system to conduct self-measurements with the devices (eg, blood pressure device) and use a tablet device. The participant sample was limited to patients of SGS Reutte. The nurses filtered suitable patients to those they thought were able to use the telemonitoring system. All eligible patients were personally asked to participate using information material about the project and were included if they provided written consent. The participating nurses were responsible for delivering the devices to the patients and for patient training on how to use the system for the field test. The nurses were educated by the system developers on how to use the prototype for both roles: the nurse role and the patient role. They also delivered the devices, educated the patients, and provided primary support for the patients during the test period.

The VITAMO test environment, including the server and connected mobile devices, was set up, and the ability to log on to the server was ensured according to the previously published implementation [11].

A basic initial working prototype was provided to start the field test. The test was integrated into the development process of the prototype (ie, a formative evaluation provided feedback for loopback changes). The feedback of participating nurses was given through email and telephone directly to the developers. All support requests were validated and ranked (ie, critical errors, standard errors, ideas for improvement), where the most critical and crucial issues got solved first. A support hotline was offered by the developers who were available on workdays between 8:00 am and 6:00 pm for technical, functional, and organizational questions and problems.

### Quantitative Evaluation

The summative evaluation included quantitative and qualitative components. For the quantitative evaluation, the server log files were analyzed according to the system’s utilization (eg, frequency of measurements, activities patients conducted with the devices), which analyzed the technical environment for stability, performance, and user interaction. Further, system logs were analyzed for the state of the mobile connection (between tablet and server) and Bluetooth connection (between tablet and Bluetooth Low Energy [BLE] devices).

To consider the related effects on the system’s uptime, further investigations were conducted to examine the available internet speed via the mobile data network and the data volume required for system operation.

The technological environment was basically analyzed through speed tests of internet data transmission and server logs. The speed tests were conducted using the online test from the Austrian Regulatory Authority for Broadcasting and Telecommunications [13] once a week for all active tablets during the test phase.

The raw logs of the server, as well as the other quantitative investigations (eg, internet speed tests), were processed and analyzed by developed R scripts (R Core Team, Vienna, Austria).

### Qualitative Evaluation

**Figure 1:**
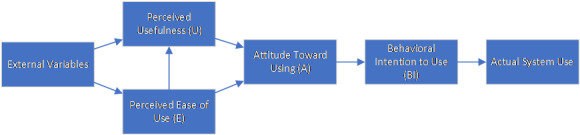
Technology Acceptance Model (TAM) [14].

The qualitative evaluation was based on the Technology Acceptance Model (TAM) [14] and focused on user satisfaction for patients and nurses (Figure 1). To date, the original TAM has been modified several times, and although the evaluation concept was created already more than 2 decades ago, it has been used for many technology evaluations [15].

Stakeholders, nurses, and patients were evaluated according to the TAM, which tries to determine the attitude towards using a certain system based on how easy the system was to use and how users perceived the usefulness of the system. Based on the perceived usefulness and the attitude towards using, a behavioral intention might be inferred. For the VITAMO evaluation, the participating nurses received a 34-question survey using a 5-point Likert scale. It included the topics as proposed by TAM: ease of use, perceived usefulness, attitude toward using, and behavioral intention to use. We added one topic for general attitudes toward information technology (IT). A specific field for “no answer” was added for each question. All TAM questions were randomly displayed to hide any topic affiliation. One additional question about the frequency of the use of VITAMO was added, with the following possible answers: several times a day, once a day, once every 2-3 days, and less than once every 3 days. Further, 2 open questions were included: ideas for VITAMO to support mobile care and general comments. The evaluation followed a triangulation approach where the survey analysis was extended with a semistructured group interview to get further information about the experiences and user acceptance during the field test. The interview guideline had 3 topic groups:

1. Technical: experience before the pilot started, experience during the pilot (eg, problems with devices, interfaces, phone calls, reminders, updates), usability (ie, pros and cons), and effects on the care workflow
2. Social: effects of the VITAMO system on the patient-nurse relationship
3. Economic: impact on work assistance, optimization of visits

The interview was qualitatively analyzed by comparing the results with the quantitative analysis as well as the surveys. Due to the participating patients’ old age and health status, a shorter survey with 7 questions focusing on ease of use and patient satisfaction and a 5-point Likert scale was conducted with the remaining 6 patients at the end of the pilot phase. This survey also included 2 open questions about the perceived pros and cons of VITAMO. The survey was distributed immediately to all participating nurses and patients after the field test ended. The raw results of all paper-based surveys were transcribed to Excel (Microsoft Corp, Redmond, WA), exported as CSV files, and analyzed using scripts implemented with R. The focus was set on a descriptive analysis. The use of boxplots for ordinal data even for the small set of participants was intended, as it provides insights into the distribution of the selected answers, which reflects the anonymous attitude of all participants. However, it should be noted, that no intercorrelation between questions is possible. All interviews and surveys were held in German, and all results were translated to English for this paper.

## Results

### Field Test

The pilot field test was held in the district Reutte, Tyrol, Austria. Reutte had ~31,000 inhabitants in 2015, and 18.6% of the residents were older than 65 years. Care allowance was received by 32.7% of persons older than 75 years. The regional mobile nursing organization in Reutte had 381 patients in 2015, with an average weekly 1.5 hours spent per patient. In that year, the nurses drove 324,774 km in total [16].

**Figure 2:**
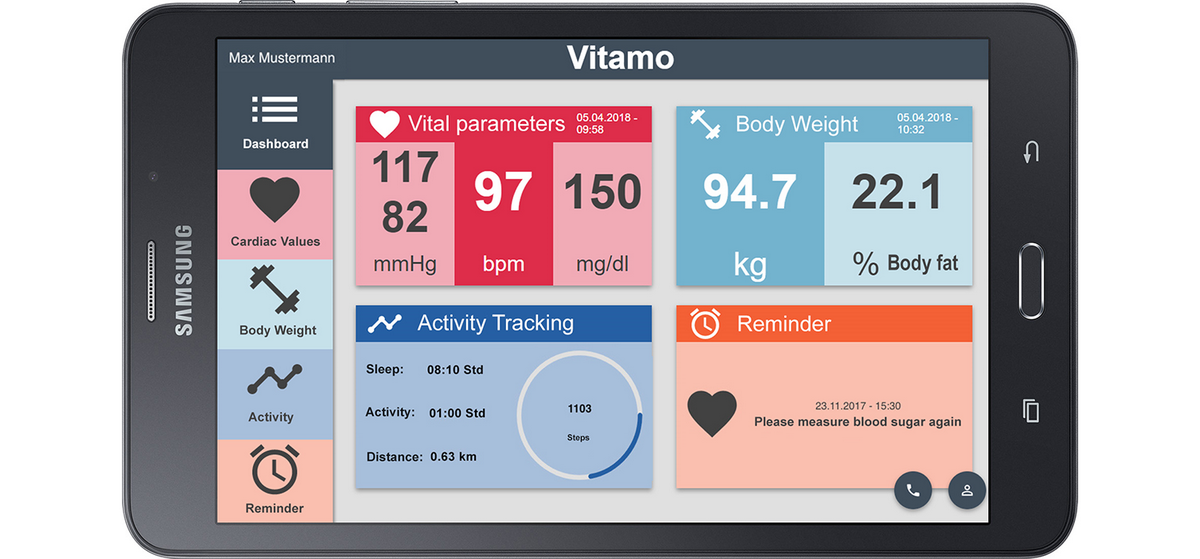
Dashboard view of the VITAl parameter MOnitoring (VITAMO) tablet app.

The pilot test was conducted from November 2017 until February 2018 (a total of 103 days). The system was being used by 8 nurses in their daily working routines. Patient recruitment was based on the nurses' experience of whether a patient would be able to self-monitor their vital signs. The nurses listed the eligible patients (28/381), and each patient was asked to participate in the project. Finally, 7 patients (4 female, 3 male; mean age 70.83 years, SD 14.13 years) were recruited to participate in the 3-month test. One patient quit after one week without giving a reason. Within the first week of the test phase, each participant received a tablet with Long-Term Evolution (LTE) mobile connection and BLE devices for blood pressure and heart rate measurements, body weight and body composition measurements, and activity and sleep tracking. A screenshot of the mobile app is depicted in Figure 2. Two people also received devices to regularly measure blood glucose. The responsible nurses introduced and educated the patients about how to use the devices and tablets at the patients’ homes. The combination of a tablet and the related devices are called a “set” for the remainder of this paper.

In total, 8 nurses participated during the test phase of VITAMO. All participating nurses and patients are part of the local mobile care organization in Reutte. In addition to the sets for the 7 participating patients, 2 additional sets were integrated into the field test (1 used by the nurses of the mobile care organization simulating a patient and 1 by the developers). The nurses’ test set was used within the production environment while the developer set was only used in the staging environment without a remote internet connection. As the study was intended to evaluate technical feasibility but not health care–related content, the nurses’ test set was also included in the summative evaluation, while the developer set was not.

### Quantitative Results

#### Usage

After the field test, a log analysis was used primarily to extract relevant values concerning direct use by patients (ie, total executed measurements and mean tablet unlocks per day) and technological aspects (ie, mean data volume and internet connection speed). The results from the log analysis, interviews, and survey were collectively analyzed.

The log analysis was conducted for measurements collected over the entire test period. The results were extracted from the server log database. Table 1 shows the total number of different measurements for each set during the test phase. The patient with set 4 ceased participating during the project and was not included within the survey but was included in the log analysis. The fitness tracker was excluded from this analysis as this device did not have an active user-triggered measurement process; the tracker was automatically synced periodically (ie, the fitness tracker device tried to connect to the tablet every 15 minutes to synchronize the data from the previous 24 hours). The tablet then managed the transfer of new data to the server for storage. Each participant was encouraged to complete at least one measurement of each type per day.

**Table 1:** The number of measurements executed for each combination of a tablet and the related devices (ie, set) over the complete 3-month pilot phase.

Set	Blood pressure	Blood glucose	Body weight	Heart rate
Set 1	189	0	113	189
Set 2	77	0	36	77
Set 3	121	86	99	121
Set 4^a^	13	6	5	13
Set 5	132	0	111	132
Set 6	120	0	120	120
Set 7	196	88	103	196
Mean of all sets	121.14	25.71	83.86	121.14

^a^The patient with Set 4 discontinued participation at the beginning of the test phase.

Information about tablet use was derived from the number of tablet unlocks. An unlock was defined as the first touch interaction with the tablet after a minimum 10-minute duration with no interaction. The VITAMO app was continuously pinned on the tablet’s screen, and no other app (nor the home screen) could be accessed on the tablet. On average, each tablet was unlocked 1.47 times a day. When interacting with the tablet, patients usually accessed the dashboard, followed by viewing blood pressure and body weight (see Table 2). Patients changed the views of the app more often than the nurses.

**Table 2:** Frequency at which different views were accessed in the VITAMO app, categorized by patients and nurses.

View	Patients (n=7)	Nurses (n=8)
Activity	116	28
Dashboard	172	135
Reminder	22	8
Blood pressure	158	47
Body weight	124	25
Total views	592	243

The frequency at which nurses opened patients‘ details decreased continuously throughout the test period, starting at 180 opens in week 1 and decreasing to 6 opens in week 14.

#### Technical Environment

The mobile internet connection was used to establish a connection between the server and tablet. The average upload volume per set during the test period was 517.3 MB (about 172 MB per month), and the download volume was 244.9 MB (about 82 MB per month), including the pilot phase. Set 4 was excluded from this analysis due to discontinued participation. A detailed view of the volume of each set is depicted in Table 3.

**Table 3:** Overall upload and download data volumes needed for each tablet and the related devices (ie, set) during the complete test period.

Set	Upload data volume (KB)	Download data volume (KB)
Set 1	221,079.44	624,484.68
Set 2	365,358.84	591,325.24
Set 3	242,408.92	670,272.28
Set 4^a^	45,576.20	64,863.52
Set 5	266,006.36	625,790.48
Set 6	233,961.16	375,895.64
Set 7	238,564.44	421,482.80
Set 8	188,388.72	398,916.04
Total data volume	1,801,344.08	3,773,030.68

^a^The patient with Set 4 discontinued participation at the beginning of the test phase.

The total data transfer volume for the test phase was 6097 MB. However, 2.5 million log entries were transmitted, which is related to a volume of 1100 MB. The mean volume for one measurement was 0.5 KB.

The test for internet speed was conducted weekly for each set, resulting in an average upload speed of 6.14 Mbps and download speed of 32.40 Mbps. The speeds for each set are shown in Table 4. Except for one set, the LTE connection of all tablets could be guaranteed continuously. Due to poor reception, one set could only partly be reached via 2G (EDGE) mobile technology, and sometimes complete loss of connection occurred.

**Table 4:** The mean upload and download speeds and pings for the internet connection of each each tablet and the related devices (ie, set).

Set	Mean download speed (Mbps)	Mean upload speed (Mbps)	Mean ping duration (ms)
Set 1	13.0	0.98	30.0
Set 2	46.0	9.18	25.2
Set 3	36.4	9.42	24.4
Set 5	45.6	5.34	25.6
Set 6	14.8	2.202	32.2
Set 7	37.0	9.24	25.6
Set 8	34.0	6.64	29.0
Mean for all sets	32.4	6.14	27.4

### Qualitative Results

#### Survey

The nurses provided the evaluation questionnaire to the patients after the pilot test ended. All remaining 6 participating patients returned the questionnaire. 

**Figure 3:**
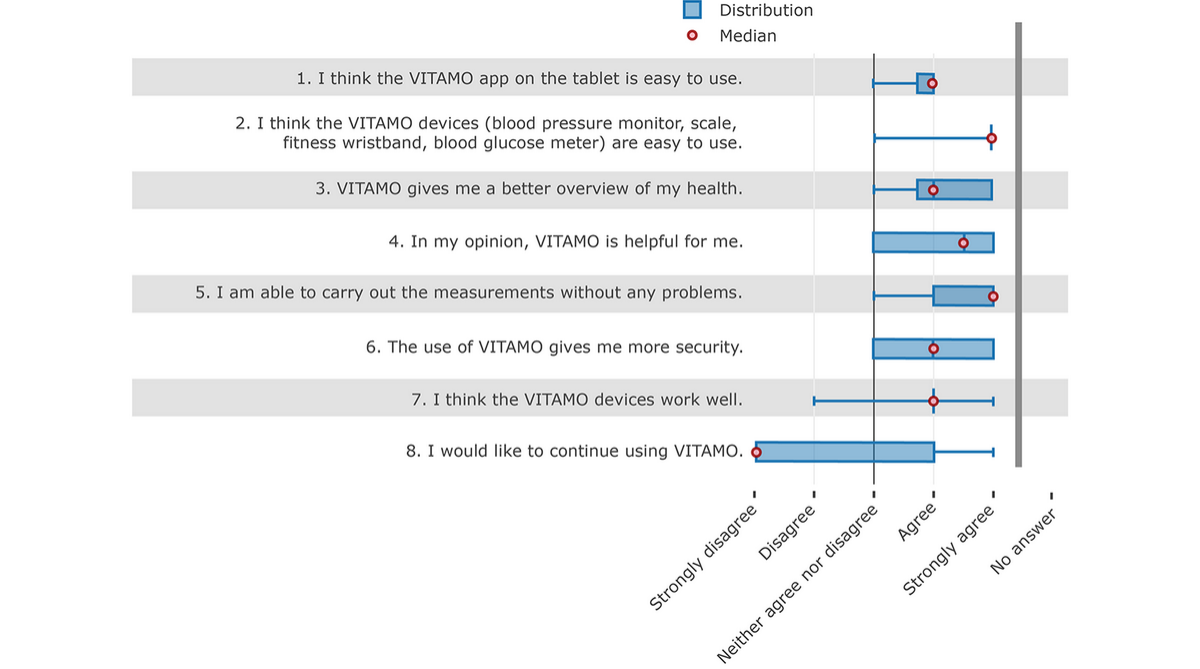
Results of the evaluation survey completed by the participating patients at the end of the pilot phase.

The qualitative evaluation of the questionnaire regarding the patient’s use of VITAMO showed that both the tablet and measuring devices were perceived as easy to use (see Figure 3). The smart devices performed slightly better than the tablet (see Figure 3, items 1 and 2).

On average, the patients also agreed that the system gives them a better overview of their health status and the system was helpful for them. The participants also stated that the system gives them more security in daily life. It is noticeable that none of the participants assessed the system negatively according to whether it aided functionality (see Figure 3, items 3, 4, and 6).

Further, there were no problems in carrying out measurements with the system. The functioning of the devices was assessed similarly, although greater distribution was observed in the responses (see Figure 3, items 5 and 7).

Although the overall rating by the patients was very positive, the primary question about further use of the system was answered negatively by the majority of patients. The boxplot shows that the answers to this question had the most spread. According to these results, no general conclusion can be derived regarding further use of the system. Patients who had indicated that they wanted to continue using the system felt safer by using the system and stated that the devices worked well. Although half of those who did not want to continue using the system found it very helpful, except for one patient, they did not feel any additional security. According to the open questions, one patient found that VITAMO was very helpful and he felt safer having the system, but he did not want to use it any longer. He also stated that the devices did not always work properly, which might have resulted from a lack of awareness of the distance limitation of BLE transmission. Nurses stated that some patients separated their tablet from the devices in different rooms. Another point that might have led to increased rejection of using the system was that some patients already owned devices for measuring vital functions they were familiar with and thus did not want to change to new devices.

The evaluation of the nursing staff questionnaire was based on the categories of TAM. Thus, the following evaluation was split into the defined TAM categories. All 8 nurse participants returned the questionnaire.

**Figure 4:**
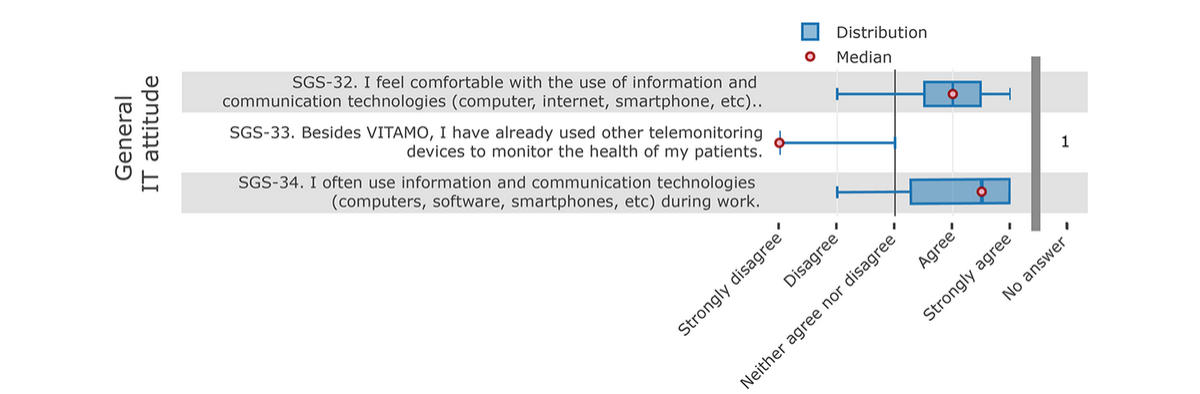
Nurses' responses to the questions about their general attitude towards information technology (IT).

The results of the questions for general attitudes toward IT show that employees were basically familiar with ICT and also used it during their work, but there was no experience with systems for vital parameter monitoring (see Figure 4).

**Figure 5:**
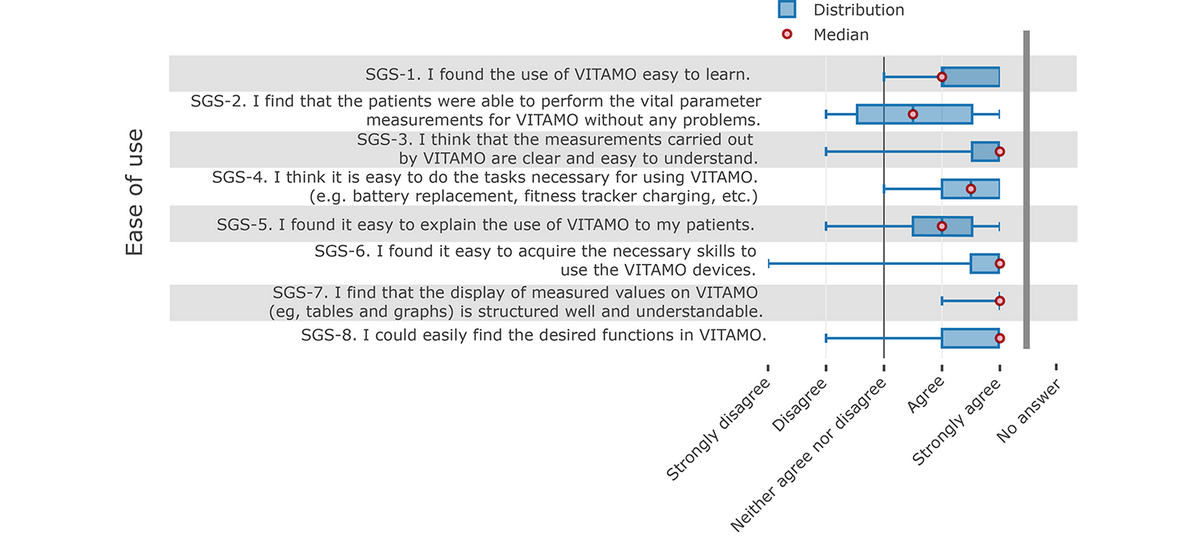
Nurses' responses about the ease of use of the system.

The results from the system’s “ease of use” questions show that VITAMO was consistently perceived as rather easy to use (Figure 5). It was easy to learn for the nurses and easy to explain to patients. The resulting vital measurements were also presented clearly, concisely, and comprehensibly. These statements also conform with the results of the group discussion (see section Qualitative Results: Survey), where the simple and clear user interface was positively rated by the nursing staff.

**Figure 6:**
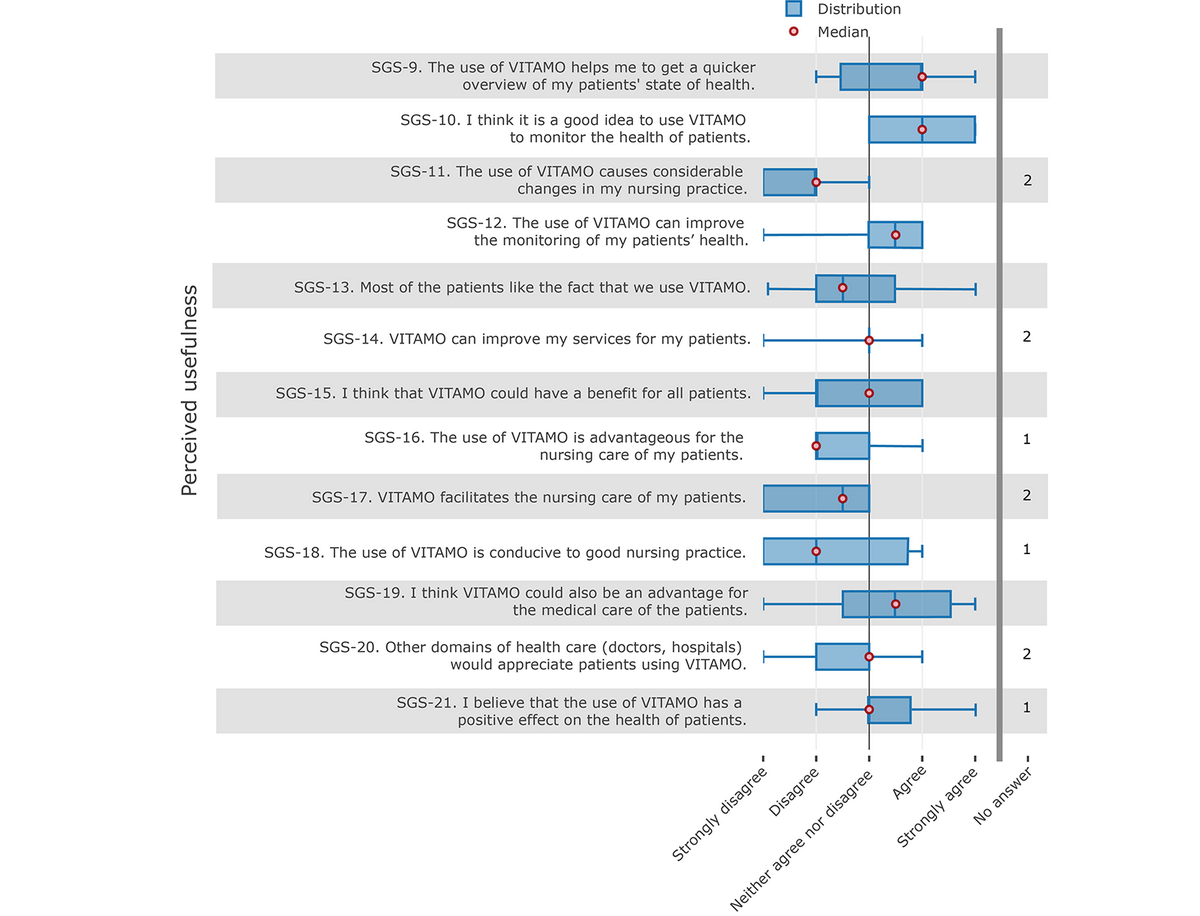
Nurses' responses about the perceived usefulness of the system.

In contrast, the analysis of the perceived usefulness showed a broader distribution of responses and generally a significantly weaker result in terms of acceptance. In particular, the answers to the topics regarding its usefulness for everyday care (see Figure 6, items SGS-11, SGS-16, SGS-17, and SGS-18) revealed that VITAMO did not bring any convincing benefits for everyday care.

There were inconsistencies in the answers to other questions. For example, nurses agreed on the benefits of getting a quicker overview of the patients’ health status or for monitoring the patients’ health status (see Figure 6, items SGS-9 and SGS-10). Concerning the direct nursing benefits for the patients, nurses’ answers were slightly negative (see Figure 6, items SGS-15, SGS-16, and SGS-17).

The number of “no information” responses selected in the “perceived usefulness” category (n=11) was also higher than for the other categories (total for all other categories, n=5).

Further results on the acceptance and usability were derived from the interview, which are reported in the next section.

**Figure 7:**
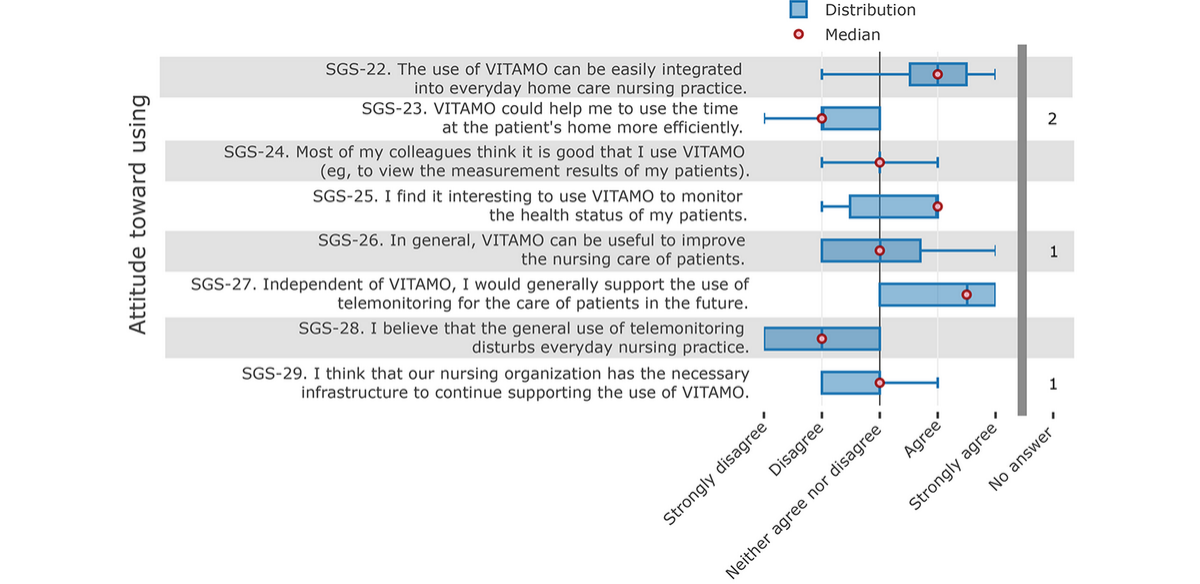
Nurses' attitudes toward using the system.

The “attitude towards using” category is influenced by perceived usefulness and the ease of use. Thus, telemonitoring is generally welcomed as a future trend (Figure 7, item SGS-27) and such a system is not regarded as a disturbance (Figure 7, item SGS-28). Furthermore, although the use of VITAMO to monitor the health status of patients is regarded as rather interesting, VITAMO could not lead to a more efficient daily nursing routine, according to the participating nurses, which supports the results from the “perceived usefulness” category.

**Figure 8:**
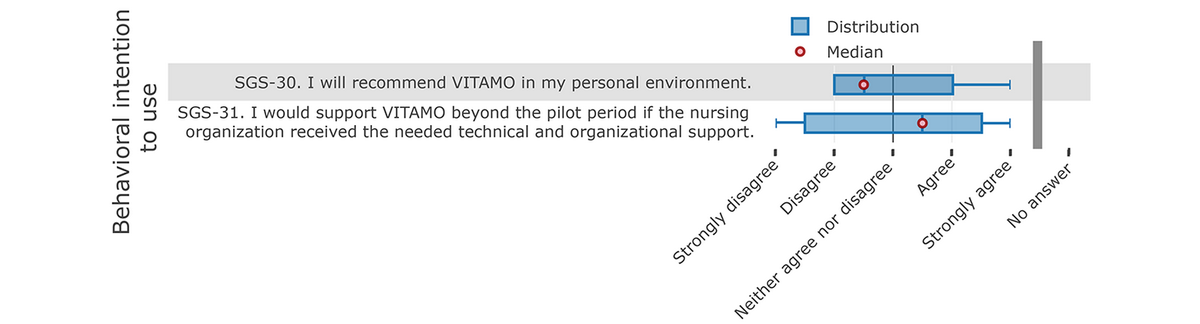
Nurses' responses about their behavioral intention to use the system.

Following the TAM approach, the “behavioral intention to use” (see Figure 8) shows a discrepancy in the answers. According to the analysis, nurses are somewhat reluctant to recommend VITAMO; however, when the nursing organization receives the needed support, the participating nurses would support the continuity of VITAMO.

#### Interview

The interview was semistructured and contained open questions, which qualitatively investigated the experiences and opinions of the nurses regarding technical, social, and financial aspects of the pilot study. Notes were taken by the interviewing author for further analysis of the answers. According to the nurses, the software on the tablet and in the web browser was straightforward to use for both nurses and patients. The graphical interface of the system was very appealing, and the graphical representations (eg, trends in blood pressure) were perceived as easy to understand. The tablet was not always easy to use. For some patients, the tiny keys on the side tablet were pressed unintentionally, and the tablet was switched off, which did not influence the overall system but created doubt for the patient on the proper use.

One patient, in particular, had problems with the capacitive touch screen because it did not react to inputs or only with difficulty. According to the nurses, this was presumably due to calluses on the fingers because for a different person it worked as intended; however, the precise reason could not be identified.

According to the nurses, patients liked the fact that they did not need much interaction with the tablet to use the system. Thus, one patient no longer used the tablet throughout the test period because the measurements were automatically transferred to the nurses without any tablet interaction.

Uncertainty among the patients was initiated by the Android update message, which was displayed on the screen at regular intervals. During the test phase, it was recommended not to install this update and to postpone it in order to not influence the proper function of the VITAMO app during the pilot phase. Some patients had doubts about doing something wrong by not installing the updates, but it was not possible to disable the Android notification.

The staff rarely had to help the patients with executing the vital sign measurements, except for the blood glucose meter, which required a higher degree of fine motor skills from the patients due to its small size (especially the small test strips). Although patients had problems closing and opening the blood pressure monitor in the beginning, assistance with measuring blood pressure was reduced over time. The fitness tracker was criticized for its cumbersome charging (the charger was difficult to mount on the device) and the poor display (brightness was too low, so that the display could rarely be read outdoors). However, proper functioning of the fitness tracker for VITAMO required no direct user interaction with the device. Therefore, the bad display quality had no effect on the measurement results. The weight scale worked without any problems to measure weight. However, the additional function for body composition analysis did not always provide results, even though all provisions were met.

According to the participants, reminders were not used for the patients due to the lack of demand, but reminders were created for the test set, which was also executed accordingly.

Training the patients for the devices was not perceived as time-consuming; all patients were already familiar with vital sign measurements before the pilot study. Due to minor problems with the equipment (especially the tablet, scale, and fitness tracker), additional visits for the patients were necessary, especially at the beginning of the pilot test.

In the interview, the nurses also criticized the limited distance of Bluetooth transmission. However, according to the requirements, the decision to use low distance transmission standards was deliberate as this is common to existing and available products, especially for wearables and smart devices with low power consumption.

The telephone function caused problems with the local IT infrastructure through firewall configurations of the nursing organization at the beginning of the pilot, and a working implementation at the workstation could only be provided at the end of the pilot phase. Thus, a proper evaluation for the telephone function was not considered. As the call was displayed on the tablet or PC, the patients were called back through regular telephone when they tried to use the call function. The actual reasons for calls were mostly related to questions regarding the VITAMO system (eg, transmission errors). According to the nurses, video telephony would also be very interesting and desirable. This function was implemented in VITAMO but could not be activated due to limitations with the transmission of telephone calls through the available internet connection.

According to the nurses, the patients were interested in the devices and conducted the measurements regularly, which confirms the log results in Figure 1. The patients wore the fitness tracker continuously during day and night except for one patient who refused to use the fitness tracker.

The pilot phase showed that incorrect measurements resulting in erroneous data transmission led to uncertainty among the patients, even if the missing measurement results were transmitted automatically or with the next measurement. According to the nurses, it would have been good to provide technical training to the patients to avoid fear (eg, when unimportant but unpreventable system messages occur). The patients also had different expectations from the pilot phase: Some assumed that the system is a final, commercial product that would work as such (eg, no erroneous transmissions), but this could not be guaranteed for this prototype implementation.

No concerns or comments were stated from the patients’ families regarding the use of VITAMO. According to the interviews, the nursing organization would also consider the integration of relatives in VITAMO to be useful (eg, to allow relatives to access vital sign measurements).

VITAMO was used by nurses to see the patient’s vital parameters during the pilot phase. The frequency of use declined over time in the pilot phase, according to the results of the log analysis. As revealed from the interview, constant monitoring of the vital parameters was not considered necessary for the participating patients due to their rather good state of health. Thus, it was not possible to characterize any additional benefit for monitoring vital parameters via mobile care. Some of the nurses discussed the values with the patients at home during their regular visits.

Some patients also took the measurement results when visiting their primary physician. Different reactions were observed from physicians, ranging from a discussion of the measurement values to a full rejection of a more detailed analysis. However, the impact of VITAMO on other caregiving providers was not part of the present study.

The nurses evaluated the developer support as very good. In addition, the VITAMO system was always accessible, and no downtimes were noticed according to the nurses, which also confirms the log analysis with a server uptime of nearly 100% and about 3 minutes downtime in general caused by the continuous app updates (no longer than seconds for one update). The measured values from the devices were not always immediately visible on the tablet (due to erroneous transmissions), but the system managed to successfully transmit all measured data, even though it was sometimes delayed. During the pilot phase, two VITAMO software updates were installed, but they were not perceived as disturbing or sometimes not even recognized at all.

According to the interviews, the measurement results would be particularly interesting for the nurses. However, the accuracy of the step and sleep activity in particular needs to be interpreted carefully, as the fitness trackers did not provide any measurement accuracy details. This was also determined via the test sets, such as when the fitness tracker recorded too many steps. Any health care–related evaluation was not part of the present study and was not considered for further analysis.

In addition to the problems with the system identified during the pilot test, the nurses provided further suggestions for improvement. A possibility for a (limited) multimedia offering on the tablet would be desirable, which could contain the daily newspaper, obituaries, or videos.

Regardless of the technical implementation of the system and pilot study, participating nurses critically noted that a more specific nursing focus of the project or the VITAMO system would have been desirable. Therefore, more exceptional nursing benefits could have been achieved. According to the nurses, a more extended project period would have been desirable, but only when more precise nursing scenarios were described.

## Discussion

### Overview

This paper investigated the technical implementation and acceptance of the telemonitoring system VITAMO, which is intended for use in rural areas and to support mobile care organizations. The focus of the present work was to plan and conduct the field test and evaluate the impact of the developed system on the mobile nursing organization.

The methodology followed the idea of using gathered requirements for creating a suitable concept for supporting mobile nursing. This concept was implemented as a prototype by adhering to defined use cases of mobile care and utilizing existing mobile technology. The implementation followed an agile approach, first developing a working prototype and using this prototype to start the field test. During development and field testing, close collaboration with nurses enabled us to continuously integrate feedback into software updates. The release cycle was determined dynamically, but new versions were usually released every 2 weeks. This agile methodology also considered the fact that further requirements arose or changed during actual use in the field. Following such an approach [12], it was possible to react quickly and efficiently to the changed requirements and continue the test with an updated and stable system. This provided benefits not only for the developer related to continuous feedback on the needs and bugs but also for the end users as they received continuous support and prompt updates.

The combination of quantitative analysis through log inspection and qualitative analysis through the TAM was used to evaluate the results from different perspectives.

The 103-day pilot phase evaluated the prototype system in a real-world environment. The sample of participants was limited to patients of the SGS Reutte. The sample of nurses (n=8) and patients (n=6) was considered sufficient to evaluate the technology environment and feasibility of the system to support mobile nursing, as the research project was not meant to quantify the general effects of telemonitoring systems but to identify the potential of leveraging ICT in the Alpine region for mobile nursing.

### Principal Findings

The evaluation of the technology environment revealed that the available mobile internet infrastructure had the potential to ensure the needed connection to the server and to share the realtime data from the vital sign measurements. For the sole data transmission of vital sign measurements, a 2G internet connection was enough. However, telephone calls and the update procedures required better internet speed. Although the data structures were designed in complex structures according to the Fast Healthcare Interoperability Resources standard [11], the emerging data volume for raw, vital signs was low, enabling real-time data synchronization over 2G.

The connection to the server via the mobile network of the tablets worked without interruptions for almost all the devices and transmission tasks in the rural region Reutte except for one tablet, which had occasional connectivity issues. This might have been caused by the automatic switching from LTE to EDGE due to low signal on LTE, which resulted in a short connection loss. However, this automatic switch at the signal borderland between reachable areas had no appreciable effects on the system as all measurements got synchronized over time, albeit not in real-time. The integration of the patient’s home Wi-Fi might be a valuable alternative to mobile internet connectivity, especially for regions with low mobile internet connectivity. This would especially be beneficial when the system might be extended with multimedia or communication services, which would result in insufficient available mobile bandwidth.

The evaluation of the patients’ utilization of the system mainly consisted of the app’s dashboard, which contains an overview of all the most recently determined vital sign measurements. The detail views on each measurement type were rarely accessed. This also conforms with the positive feedback about needing very little interaction with the system to use it. Vital sign measurements were executed by the patients at least once a day throughout the entire test period. Based on these results, VITAMO seems to create patient awareness of monitoring vital signs, and the patients welcomed the system as long as it integrated unobtrusively in their existing environment and daily life. VITAMO was developed as a mobile system because the requirements analysis revealed the advantages of such an approach [10]. The evaluation showed that a strength of the system was the uncomplicated system maintenance and management. As stated by the users, the system offered a convenient user interface through the tablet; however, if one was not interested in such information, the system did not differ from conventional vital parameter monitoring systems or devices. Thus, ambient assisted living systems that were obtrusively integrated into one’s home were not considered as comparative approaches [17]. This also affirms the chosen system design, which is based on the idea of a progressive web app for both desktop and mobile platforms in combination with BLE devices [11]. The developed prototype followed the idea of always retaining a stable state; for example, with internet connection loss or Bluetooth transmission errors, measurement data were stored on the related devices and re-sent at the next successful connection. This behavior was recommended for the given setting, as the logs from the field test revealed occasional connection breaks.

The results of the nurses’ and patients’ attitudes towards VITAMO were predominantly positive, particularly regarding the ease of use and overall usefulness of VITAMO. The tablet was recognized as user-friendly, and the overall opinion of it was positive, even for conceivable future developments such as extending the platform with additional offers and services (eg, daily newspapers, multimedia). The usability findings agree among the different evaluation channels (ie, log analysis, survey, interview).

Despite the overall positive general and technological evaluation, patients and nurses were not convinced to keep using the VITAMO system. Regarding the reason for patients to respond this way, we made assumptions based on the evaluation findings. The participating patients already had devices to measure vital parameters (eg, blood pressure measuring device), and no further benefit of automated data transfer of the measurement results was recognized from the patient’s point of view. Further, only patients who were able to self-measure vital signs were recruited. This means the participants must have been of good enough health to operate the devices (ie, to put on the blood pressure cuff and start the device). It should be noted that this might bias the results, as participating patients were in a health state where telemonitoring might not reveal a benefit, while people in need of the solution might benefit from it to a higher extent. Log analysis of the server records and survey results with the nurses revealed that the nurses’ frequency of system use declined over time. The rather low utilization of the system by nurses might also have affected the patients’ attitudes. Apart from self-monitoring of the vital parameters, the project did not include any other scenario such as in the context of using the data for direct medical treatment when visiting a physician. The nurses also expressed that they did not see any direct nursing benefit for themselves (eg, support in their daily work) regarding the continuous recording of patients’ vital parameters. To identify the reasons for this attitude, a group discussion was conducted with the nurses. The discussion revealed that the nursing processes were very operational depending on the patient’s needs and did not follow a strategic plan. The immediate reaction to unusual measurement results in terms of an alert was deliberately not followed because the developed prototype was not related to a medically certified product. However, the nurses were able to identify any abnormal results including the abstinence of measurements but this was not used to adapt the nursing process during the pilot test. The processes of the nursing organization did not comply with any clinical guidelines for vital sign measurements; therefore, no standardized process was utilized. As a limitation of the present study, the organizational nursing processes regarding the direct interaction between patients and nurses were not deeply analyzed. Further organizational issues occurred such as the utilization of vital signs for medical purposes, as some physicians declined to use the results. Future efforts would need to integrate health-related third-party stakeholders like physicians and relatives to infer a medical benefit. Any medical decision-making was intentionally excluded from this study because the study was intended to investigate the technical feasibility and its impact on mobile care. To use the results to investigate the medical impact on patient outcomes, a bigger sample size is necessary.

The evaluation showed that both patients and nurses recognize the potential of telemedical applications in mobile home care. They also found the technical prototype implementation to be useful in mobile care. Various other projects are reporting valuable benefits on patient outcomes and reduced cost through the use of telemonitoring systems [18-21]. Further investigations of at-home telemonitoring applications have revealed reduced hospital admissions, length of stay, and mortality rates [22].

The evaluation results of VITAMO showed that the home care organization tends to be open to technology support and telemonitoring is perceived as easy to learn and use. However, meaningful use or importance of overcoming future critical developments was not recognized. This is also reflected in the weekly patient access statistics and interview results. Besides, some of the functions such as the reminder function were only used for some tests but not included in real scenarios, although requirements engineering revealed the need for such a feature. This further revealed an inconsistency in the first set of use cases of the project that were motivated through the established nursing processes.

To fully evaluate the acceptance of a telemonitoring system such as VITAMO, users (patients and nurses) need to be aware of the organizational progression and its effects on the care processes. Recent studies have highlighted the essential requirements for supportive telemonitoring ICT to adapt to existing processes [23,24]. However, the results of this study prove the necessity of establishing a compact structure of organizational processes and their aims. These findings could contribute to further investigations on care delivery processes to evaluate the effects on adapting and supporting such processes with ICT.

### Conclusions

The present study proves the technical feasibility and user acceptance of using telemonitoring to support mobile nursing even in rural regions such as the Alpine region. The critical impact of demographic changes on the need for home care has already been investigated through several research approaches, and technology has the potential to provide the necessary environment to develop meaningful solutions. Related solutions for mobile care need to leverage new technologies for both hardware (eg, the Internet of Things) and software (eg, progressive web apps). However, the intended stakeholders need to have well-organized, fully aware strategic and interconnected processes to benefit from such ICT solutions in the long term.

## Abbreviations

BLE: Bluetooth Low Energy
ICT: information and communication technology
INTESI: Integrated territorial strategies for Services of General Interest
IT: information technology
LTE: Long-Term Evolution.
SGI: service of general interest
TAM: Technology Acceptance Model
VITAMO: VITAl parameter MOnitoring
